# Molecular epidemiology of KPC-producing *Klebsiella pneumoniae* clinical isolates in hospitals in North-Western Tuscany

**DOI:** 10.1186/1753-6561-5-S6-P296

**Published:** 2011-06-29

**Authors:** A Buzzigoli, P Valentini, M Minacori, F Guarneri, R Banducci, C Tascini, F Menichetti, GM Rossolini, B Casini, GP Privitera

**Affiliations:** 1 Experimental Pathology, M.B.I.E., University of Pisa, Pisa, Italy; 2 Hygiene and Epidemiology, Azienda Ospedaliera Universitaria Pisana, Pisa, Italy; 3 Infectious Diseases, Azienda Ospedaliera Universitaria Pisana, Pisa, Italy; 4 Molecular Biology, University of Siena, Siena, Italy; 5 Hygiene and Epidemiology, University of Pisa, Pisa, Italy

## Introduction / objectives

The emergence and spread of carbapenem-resistant *Enterobacteriaceae* represents a major clinical and public health challenge, especially concerning those organisms that produce *Klebsiella pneumoniae carbapenemase* (KPC)–type b-lactamase.Â In this study, we examined the molecular epidemiology of KPC-producing *Klebsiella pneumoniae* strains, isolated during sporadic or epidemic cases in different hospitals of North-Western Tuscany (NWT).

## Methods

Starting from April 2010, an outbreak of clinical infection caused by multiresistant *Klebsiella pneumoniae*, occurred in the UniversityÂ Hospital of Pisa and in other hospitals from the surrounding area; during this period we isolated 57 *Klebsiella pneumoniae* strains in our hospital and 23 from other health-care facilities; all strains, isolated mostly from different patients hospitalized over protracted periods of time, showed reduced susceptibility to carbapenems and were assayed for KPC detection.

## Results

60 out of 61 tested strains produced KPC type 2; molecular typing results indicated the spread of a prevalent clone during the outbreak in Pisa. The PFGE method allowed to identify 7 variants clonally related (pt A1-A7), sharing the same MLST sequence type (ST 258).

## Conclusion

Molecular characterization confirmed the higher discriminatory power of PFGE compared to MLST and it may be useful in local and short-time outbreaks; however MLST, providing unequivocal and comparable data, remains the gold standard for molecular typing.

## Disclosure of interest

None declared.

